# An unusual bifid first metacarpal

**DOI:** 10.4103/0019-5413.61726

**Published:** 2010

**Authors:** Suresh Kumar

**Affiliations:** Department of Orthopaedics, Maharishi Balmiki Hospital, Pooth Khurd, Delhi 110039, India

**Keywords:** Bifid first metacarpal, swan neck deformity, thumb

## Abstract

Bifid first metacarpal is a common congenital anomaly. Here, we report an unusual case of bifid first metacarpal in a 13-year-old girl who presented with swan neck deformity of left thumb, a bony prominence on ulnar aspect of left thumb in the first web space and a bifid first metacarpal lacking its own epiphysis. The patient underwent surgery, resulting in complete functional recovery as well as cosmetic improvement of the left thumb.

The bifid thumb represents a complete or partial duplication of the thumb (preaxial polydactyly). The cause of the bifid thumb is not known.^1^ Experimental evidence suggests that any agent causing a temporary growth disturbance between the mesoderm and the ectoderm of preaxial limb bud during early maximum cell proliferation may result in a bifid thumb.^2^ The syndactyly is more common than polydactyly, and radial polydactyly is more common than the ulnar polydactyly.^3^ The appearance of the duplicated thumb depends on the changes in the skeletal and soft tissue structures, which vary with the level of the bifurcation and the extent of the duplication.^4^ The extent of the thumb duplication is extremely variable, which may be classified into 5 main types as described by Wassel. In type E, there is duplication of the proximal and distal phalanges of the thumb and splitting of the distal end of the metacarpal. There is a marked difference in the site and function of the two thumb parts because of the duplication of soft tissues.^5^ Bifid metacarpal and phalanges with their own epiphysis (duplication of metacarpal and phalanges) with or without congenital anomalies have been reported.^6^,^7^

This report describes a case of bifid first metacarpal with the bifid “metacarpal limb” that lacks its epiphysis, associated with ulnar duplication and swan neck deformity of left thumb, which is, in itself, a unique entity. There was no duplication of soft tissues or any other congenital anomaly. The deformity of left thumb got corrected after excision of ulnar duplication. Roentgenogram of the hand is essential pre-requisite to determine the level of bifurcation. A thorough search of the literature revealed that such a case has not been reported in English language literature till date.

## CASE REPORT

A 13-year-old girl presented to the outpatient department in March 2005 with complaint of bony prominence in the first web space on ulnar aspect associated with deformity of the left thumb. There was no family history of polydactyly and any other congenital anomaly. The parents gave a history of partial flexion of interphalangeal joint and nodular bulge on the ulnar aspect of left thumb since birth, which had gradually increased in size.

On examination, there was a hard bony prominence on ulnar aspect of the left thumb in the first web space. It was more prominent on dorsal aspect as compared to the palmar aspect. The thenar crease was more prominent and deep on radial aspect of hand. Flexion deformity of interphalangeal joint and hyperextension of metacarpophalangeal joint was noted, which were partially correctable on adduction of thumb and deformity became more prominent on abduction of thumb.

**Figure 1:**
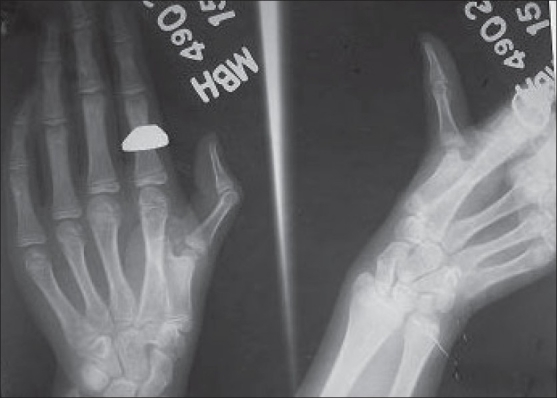
Anteroposterior and oblique radiograph of left hand including thumb showing bifid first metacarpal without its own epiphysis with hyperextention at metacarpophalangeal joint and flexion at interphalangeal joint

Roentgenogram showed incomplete bifid first metacarpal on ulnar aspect with hyper extension at metacarpophalangeal joint and flexion at interphalangeal joint of thumb. Metacarpophalangeal and interphalangeal joints were normal [Figure 1]. A clinico-radiological diagnosis of Wassel's type 5 deformity with swan neck deformity of left thumb was made.

### Operative procedure

The patient was placed in supine position with the hand kept over the hand rest. Under brachial block and tourniquet, an incision was made along the lateral aspect of first metacarpal and reached to the ulnar component of bifid metacarpal. Adductor pollicis muscle was tight and soft tissue was felt on the ulnar aspect of the bifid metacarpal. Pulling onto this soft tissue worsened the flexion at interphalangeal joint, which suggested it to be flexor pollicis longus. Normally, the flexor pollicis longus passes between the thenar muscles and the adductor muscles. The swan neck deformity of the thumb was seen to be due the action of adductor pollicis muscle on the base of proximal phalanx of thumb (causing thumb adduction), flexor pollicis longus causing flexion of interphalangeal joint secondarily leading to hyperextension of first metacarphalgeal joint due to tightness of extensor pollicis longus muscle. After excision of bony ulnar component of bifid metacarpal at the base of bifurcation, the swan neck deformity of thumb got corrected automatically due to unhindered excursion of flexor pollicis longus and adductor pollicis muscle. After surgery, the balance between the thumb flexors and extensors got restored and the swan neck deformity got corrected.

**Figure 2:**
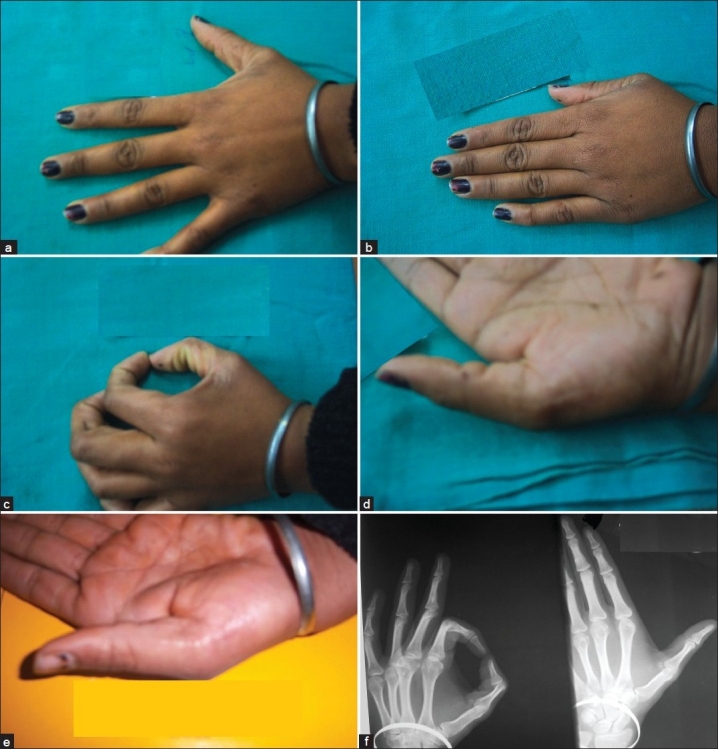
(a-e) Clinical photograph of left hand at 3 years follow-up showing old healed surgical scar on radial aspect of first metacarpal, without any bulge in the first space and normal range of motion of the left thumb (Figure 1 and  2 a-e). (f) Final radiograph at end of 3 years, showing healed excised part of bifid first metacarpal and normal interphalangeal and metacarpophalangeal joints

Stitches were removed on the 12^th^ day of surgery. The patient was then followed up clinically and radio-logically at 6-monthly intervals for 3 years to assess the range of motion at interphalangeal and metacarpophalangeal joints. At the final follow-up of 3 years, the deformity is well corrected with no residual or recurrent bulge in the first web space [Figure 2a–d]. The patient had no apparent functional impairment and was satisfied with the cosmetic result [Figure 2e]. The final roentgenogram of thumb at the end of 3 years shows normal interphalangeal and metacarpophalangeal joints with healed site of excised bifid first metacarpal [Figure 2f].

## DISCUSSION

Duplication of digits, or polydactyly, is a common and conspicuous hand anomaly. It was recorded in biblical literature as long ago as 3000 years and approximately 9000 to 10,000 new cases are recorded every year.^1^ Our case of bifid first metacarpal presented with swan neck deformity of left thumb due to the disruption of balance of flexors and extensor forces acting across the joint and was not associated with other congenital anomalies or polydactyly. The deformity got corrected automatically after excision of bifurcated metacarpal.

The pre-axial polydactyly (bifid thumb) is either complete or partial duplication of the thumb. It is most common duplication in white and oriental population, occurring in 1:3000 births. It is usually unilateral.^7^ Only 9 out of 70 patients in Wassel's series had bilateral involvement. The cause of bifid thumb is unknown.^6^ Wassel described in his classification the type 4 entity as complex duplication of distal and proximal phalanges with bifurcation of metacarpal and type 5 for complete duplication of the distal and proximal phalanges and metacarpal.^6^ Ellis-Van Creveld syndrome is a skeletal dysplasia with an incidence of approximately 1 out of 1,50,000 live births.^8^ The post axial polydactyly of hands is seen in all the patients. Polydactyly may be just extra-soft tissue not adherent to the skeleton and devoid of bone, cartilage, joints, or tendon, or the digits may show duplication with components like bifid metacarpal or there may be a complete digits formation with its own metacarpal.^4^

In our case, pathophysiology of swan neck deformity is explained by disruption of the balance of flexors and extensor forces acting across the joint. Flexor-overpull on the bifurcated metacarpal also increased the pullover the metacarpophalangeal joint. Constant efforts to extend the thumb against this pull then lead to stretching of the collateral ligament and the volar plate at the interphalangeal joint. Bifurcated first metacarpal was lacking its own epiphysis, which was congenital in origin. With this deformity, the patient was unable to perform the routine activities of the daily living. Hence, surgical intervention was necessary at early age.
